# Exhaustive feature-subset search for data-driven post-correction of satellite atmospheric processors: a case study on chlorophyll-a retrieval in inland reservoirs

**DOI:** 10.3389/frai.2026.1947948

**Published:** 2026-09-30

**Authors:** Victoria Amores-Chaparro, Fernando Broncano, Jesús A. Torrecilla-Pinero, Aurora Cuartero

**Affiliations:** 1 Department of Computer Systems and Telematics Engineering, School of Technology, Universidad de Extremadura, Cáceres, Spain; 2 Department of Computer Systems and Telematics Engineering, School of Industrial Engineering, Universidad de Extremadura, Badajoz, Spain; 3 Department of Construction, School of Technology, Universidad de Extremadura, Cáceres, Spain; 4 Department of Graphical Expression, School of Technology, Universidad de Extremadura, Cáceres, Spain

**Keywords:** chlorophyll-a retrieval, exhaustive feature selection, machine learning, out-of-distribution generalization, Sentinel-2, stability selection

## Abstract

Satellite retrieval of chlorophyll-a (Chl-a) is central to monitoring eutrophication in inland reservoirs, yet the atmospheric-correction processors it depends on, trained on oceanic and large-lake conditions, are systematically biased over small water bodies. A data-driven post-correction layer is increasingly used to reduce this bias; for such a layer over the joint output of the C2-Net processors (C2RCC, C2X, and C2XC), we ask which engineered features robustly carry the corrective signal rather than chasing an accuracy headline. Enumerating all 2^17^−1 = 131, 071 subsets of a symmetric 17-feature pool exposes a broad performance plateau on which three mainstream heuristics pick inequivalent “best” subsets at near-identical error, so any single optimum is an artifact of selection on the cross-validated statistic. Stability selection instead isolates a small robust core (led by $\sqrt{\left|\text{C}2\text{X}{\text{C}}_{r}\right|}$, chosen in 98% of subsamples at *k* = 6), the transformations that dominate the lowest-error subset being selected near chance. On 252 satellite–*in-situ* pairs from five Guadiana-Basin reservoirs (Spain), the correction improves pooled RMSE over raw C2RCC modestly but, for the Huber model, significantly (~10–16%, *p* = 0.047 under a cross-validation-corrected test), with the three estimators statistically indistinguishable from one another; under Leave-One-Reservoir-Out validation the degradation runs from ~15% (pooled) to ~34% (per-reservoir mean), both within the wide bootstrap intervals and thus approximate rather than precise. We distill a compact reporting template: an RMSE-vs.-*k* curve with variance bands, stability selection in place of a single “best” subset, a relative-error metric (MdAPE) alongside RMSE, and LORO as a minimum transferability check. All code, data, and models are released openly.

## 1 Introduction

The accuracy and operational use of machine-learning systems for environmental sensing increasingly depends on how well a model trained in one setting transfers to another (Ratte-Fortin et al., 2026; Kumar et al., 2025). The difficulty is particularly acute when a pre-trained component partially captures the underlying physical signal, and a downstream learner is asked to correct its residual biases. This two-stage data-driven post-correction is now common in satellite remote sensing of water quality, climate downscaling, and atmospheric sciences (Tran et al., 2025; Wu et al., 2025; Huang et al., 2026; Nguyen et al., 2026), but the feature-engineering and validation choices that follow remain under-examined relative to end-to-end approaches.

The retrieval of chlorophyll-a (Chl-a) in inland reservoirs from satellite imagery is a representative instance of this problem. Eutrophication of freshwater systems is one of the most widespread environmental challenges of the present decade (Khan and Ansari, 2014; Sòria-Perpinyà et al., 2019; Joffre et al., 2026), threatening drinking-water supplies and triggering regulatory monitoring obligations such as the European Water Framework Directive (European Parliament and Council of the European Union, 2000). Conventional *in-situ* sampling is unable to meet the temporal and spatial frequency required by these programs, especially in semi-arid Mediterranean regions where a single basin can include dozens of reservoirs of widely varying size (Richardson, 1996; Cuartero et al., 2023; Cáceres-Merino et al., 2024). Multispectral satellite missions such as Sentinel-2 (MSI) provide the data needed for systematic monitoring (Drusch et al., 2012; Zeng et al., 2023): Sentinel-2 is a multispectral, not hyperspectral, sensor with 13 discrete bands between 443 and 2,190 nm at native resolutions of 10, 20, and 60 m. The link between top-of-atmosphere radiance and Chl-a is non-trivial because atmospheric correction is required first, and Chl-a then has to be inferred from optically complex Case-2 water reflectances (Schroeder et al., 2022; Vanhellemont and Ruddick, 2021; Sanchez-Perez et al., 2026). For this purpose, the European Space Agency provides the C2-Net family of neural-network atmospheric correction processors (C2RCC, C2X, and C2XC), each trained on different radiative-transfer regimes (Doerffer and Schiller, 2007; Brockmann et al., 2016; Soriano-González et al., 2022). While C2-Net processors are widely used, several studies have documented substantial biases when they are applied to small inland reservoirs whose optical conditions, adjacency effects, and trophic dynamics differ from those in the training regime (Cuartero et al., 2023; Cáceres-Merino et al., 2024; Ansari et al., 2025).

A growing body of work proposes machine-learning corrections to satellite-derived water quality estimates (Pahlevan et al., 2020; Saberioon et al., 2020; Wang et al., 2022; Santos et al., 2024; Jeong et al., 2025; Nguyen et al., 2026). Compared with end-to-end retrieval from spectral bands, the *post-correction* paradigm (Tran et al., 2025; Xiong et al., 2025; Niroumand-Jadidi et al., 2021) retains the physical information embedded in the atmospheric processor outputs and treats the learning problem as one of bias correction. The same logic has been successfully applied in adjacent domains, for example, neural-network bias correction of SMAP sea-surface salinity retrievals (Wu et al., 2025), making the paradigm a natural fit for inland-water Chl-a as well. Our group has recently demonstrated a closely related post-correction strategy on a broader sample of 32 small reservoirs in Extremadura, achieving up to 35% RMSE reduction relative to the best C2-Net baseline (Torrecilla-Pinero et al., 2026), extending an earlier proof-of-concept for the same problem (Amores-Chaparro et al., 2026). The present study complements that broad-coverage work with a depth-coverage focus (five reservoirs, ~50 matches each), enabling exhaustive feature enumeration and explicit out-of-distribution validation. We emphasize that the resulting model is data-driven: the physics enters the system only via the upstream C2-Net processors, not through a physical prior on the learner itself.

Within this paradigm, two methodological questions are seldom addressed systematically. The first concerns feature engineering. The atmospheric processors produce a small number of outputs that can be combined into a much larger pool of nonlinear transformations and interaction terms. Which subset of features yields the lowest cross-validated error, and how robust is that subset to the choice of search procedure? Heuristic wrapper methods (forward sequential selection, recursive feature elimination) and filter methods (mutual information) are the standard tools (Guyon and Elisseeff, 2003; Hutter et al., 2019), yet they are rarely benchmarked against an exhaustive baseline. The second concerns the metric basket. Most studies report a combination of RMSE, MAE, *R*^2^, and MBE; whether these metrics capture orthogonal aspects of model behavior on real datasets is rarely tested.

This study's central question, developed through a worked case study of a 252-pair satellite–*in-situ* dataset from five reservoirs in the Guadiana Basin (Spain), is which engineered features robustly carry the corrective signal, and whether a correction built on them works. We construct a symmetric 17-feature pool from the three C2-Net processor outputs and enumerate all 2^17^−1 = 131, 071 subsets under a controlled pipeline, comparing the exhaustive optimum with three heuristic selectors (forward sequential, recursive feature elimination, mutual-information ranking). The heuristics select inequivalent subsets with near-identical error, revealing a plateau where any single choice is arbitrary. Because selecting the single lowest-error subset from 131, 071 candidates on one dataset is a textbook selection-on-the-statistic (“winner's curse”) setup, we do not attach meaning to any individual optimum; instead, we apply stability selection across resamples to identify the features that are robustly selected (and therefore carry the signal) rather than interchangeable within the plateau. We then confirm that a correction based on the parsimonious feature set performs well, as evidenced by per-reservoir performance, a comprehensive metric basket, and Leave-One-Reservoir-Out (LORO) out-of-distribution validation (Radocaj et al., 2025; Nourmohammadi et al., 2025). A secondary methodological observation concerns metric redundancy: across 1,500 evaluated subsets, only MdAPE adds a non-redundant, relative-error axis to the usual RMSE, MAE, *R*^2^, and kurtosis battery. Together, these motivate a compact reporting template we suggest for post-correction studies in this area. The case study yields a modest pooled gain over raw C2RCC (~10%–16% depending on aggregation, statistically significant for the Huber model at *p* = 0.047 under a cross-validation-corrected test, though the three estimators are indistinguishable from one another) and a residual structure at one reservoir (Zújar) that we treat as a candidate sample-level out-of-distribution case rather than a method failure.

## 2 Related work

### 2.1 Methodological background: feature selection and spatial generalization

The feature-selection literature recognizes three families of algorithms (Guyon and Elisseeff, 2003; Hutter et al., 2019): filter methods that rank features by univariate statistics, wrapper methods that search over subsets using a base estimator, and embedded methods that fold selection into the estimator. Modern AutoML combines these with hyperparameter optimization (Feurer et al., 2015; Olson et al., 2016), and recent automated feature-engineering work on environmental tabular data has begun to ask which solution a given algorithm actually returns (Dai et al., 2026; Kraus and Bartsch, 2026; Khatiriolyaee et al., 2026). Exhaustive enumeration is rarely used because the search space grows as 2^*p*^, but for small pools and modest datasets it is feasible and useful: it yields the optimal subset under the chosen protocol and exposes whether cheaper heuristics converge to it. We adopt it as a diagnostic, not as a proposed selection method. The parsimony pattern we report is not specific to water quality: in an adjacent remote-sensing domain, (Alam and Simic Milas, 2025) compress a 64-dimensional feature space for canopy chlorophyll, nitrogen and phosphorus retrieval and likewise find that accuracy saturates once a small, information-rich subset is retained.

Conventional cross-validation can overestimate accuracy on spatially correlated environmental data, because random splits leave nearby points in both training and test partitions (Radocaj et al., 2025; Nourmohammadi et al., 2025). Spatial cross-validation holds out a region rather than a random sample; Leave-One-Region-Out and similar protocols are now standard sanity checks on the transferability claims of geospatial models (Ratte-Fortin et al., 2026; Xu et al., 2026). In the inland-water context, the natural region is the reservoir, so LORO directly simulates deployment to unmonitored water bodies. The broader out-of-distribution literature in remote sensing (Al-Emadi et al., 2025; Wang et al., 2026; Kumar et al., 2025) frames this as covariate drift between training and deployment, the lens under which we later analyze the Zújar case.

### 2.2 Atmospheric correction context: Sentinel-2, C2-Net, and post-correction

Sentinel-2 carries the Multispectral Instrument (MSI), a multispectral (not hyperspectral) sensor with 13 bands between 443 and 2,190 nm at 10–60 m resolution depending on band (Drusch et al., 2012). Within ESA's Sentinel Application Platform (SNAP), atmospheric correction over Case-2 waters is provided by three sister processors that differ in training regime rather than network architecture: C2RCC, the standard, trained on a balanced range of optical conditions (Doerffer and Schiller, 2007; Brockmann et al., 2016); C2X, whose envelope extends toward extreme conditions (high CDOM, high suspended sediment); and C2XC, designed for intermediate inland and coastal waters (Soriano-González et al., 2022). Recent independent evaluations report large discrepancies between them depending on the optical regime (Sanchez-Perez et al., 2026; Cuartero et al., 2023; Cáceres-Merino et al., 2024; Ansari et al., 2025). Table 1 summarizes their characteristics as used here.

**Table 1:** The three C2-Net atmospheric correction processors compared in this study.

	C2RCC	C2X	C2XC
Design intent	Standard Case-2 waters with moderate optical complexity	Extreme optical conditions (high CDOM, suspended matter)	Intermediate inland and coastal waters
Training regime	Balanced range of inherent optical properties (IOPs)	Extended IOP envelope toward extremes	Intermediate range, biased toward inland waters
Salinity default	0.5 PSU (freshwater preset used here)	0.5 PSU	0.5 PSU
Reported behavior on small inland reservoirs	Most reliable baseline; mild over-estimation (Cuartero et al., 2023)	Frequently amplifies error at high Chl-a (Cáceres-Merino et al., 2024)	Highly variable; best suited to intermediate optical regimes (Sanchez-Perez et al., 2026)

These known biases motivate a broader paradigm in which a physical model is paired with a data-driven correction layer rather than discarded (Fink et al., 2026; Huang et al., 2026). The empirical-vs.-hybrid trade-off within this paradigm is itself an active question: Alam et al. (2025) compare purely empirical machine-learning regressors against the same regressors coupled to a PROSAIL radiative-transfer model for canopy biochemical retrieval, the direct analog in vegetation remote sensing of the physical-model-plus-correction design pursued here. Examples in adjacent domains include physics-informed bias correction of satellite salinity retrievals (Wu et al., 2025), observation-informed convective parameterizations (Huang et al., 2026), and bias-correction pipelines for satellite Chl-a in turbid waters (Tran et al., 2025; Xiong et al., 2025; Nguyen et al., 2026), whereas Naser (2026) adds a cautionary note about physics-informed neural networks whose physical priors are weakly constrained. The most directly comparable work is the recent companion study by Torrecilla-Pinero et al. (2026), which applied a closely related post-correction strategy (rescaled processor outputs, interaction terms, aggregate statistics, regularized regression, and gradient boosting) to a broader sample of 32 small reservoirs in Extremadura with 94 Sentinel-2 scenes, reporting up to 35% RMSE reduction over the best baseline processor. That study established the paradigm and its accuracy headline on a breadth-first design, whereas the present work is deliberately depth-first and disjoint from it in question rather than merely in sample. Its standalone contribution is methodological and does not appear in the companion study: (i) an *exhaustive* enumeration of all 2^17^−1 subsets of a symmetric feature pool, used to audit the heuristic selectors that studies in this area actually deploy, together with stability selection to identify which features robustly carry the signal once the winner's-curse artifact of picking a single optimum is removed; (ii) an empirical test of which of seven commonly-reported metrics are non-redundant on real data; and (iii) LORO as an explicit out-of-distribution stress test with the honest finding, reported below, that the apparent spatial transferability is weaker than an unweighted-mean aggregation suggests. The depth-first design (5 sites, 252 matched pairs, and ~50 pairs per site) is what makes (i)–(iii) feasible, none of which were the focus of the broader-coverage study. The post-correction layer therefore sits on the conservative end of the hybrid-modeling spectrum, leaving the physical component (C2-Net) untouched and applying the correction at the output level by a standard regressor without physical priors.

## 3 Materials and methods

### 3.1 Study area and data collection

The Guadiana River basin in southwestern Spain provides the setting for this study (Figure 1). Five reservoirs were selected to span a representative range of surface areas, storage capacities, and trophic regimes (Cuartero et al., 2023). Alange (50.4 km^2^, 852 hm^3^) and Orellana (50.8 km^2^, 808 hm^3^) are the largest. Tentudía (0.68 km^2^, 4.9 hm^3^) is the smallest. Peña del Águila at Villar del Rey (17.8 km^2^, 120 hm^3^) and Zújar (14.5 km^2^, 309 hm^3^) are intermediate. By sample-period mean Chl-a, Alange, and Orellana fall in the mesotrophic range, whereas Tentudía, Villar del Rey, and Zújar are eutrophic and exhibit episodic hypertrophic events with maximum Chl-a reaching 133–200 μg/L (Table 2). The matched pairs span all 12 months over 3 years (2023–2025; 78, 103, and 71 pairs, respectively), with balanced seasonal coverage (53–87 pairs per season) and seasonal median Chl-a of 5.5–8.7 μg/L. Hypertrophic conditions (>25 μg/L) are genuinely episodic, occurring in 8.7% of samples and concentrated in winter and summer, so the eutrophic sample-period means of the three smaller reservoirs reflect the authority's documented bloom episodes captured within a seasonally representative window rather than an unusually bloom-heavy sampling period. This range and its skew toward a few extreme events are precisely the operational conditions a monitoring correction must handle, but they also bound the results: three sites and ~50 pairs each cannot represent the full diversity of inland-water optical regimes, and the transferability of the framework beyond this range is quantified only by the LORO analysis of Section 4.9.

**Figure 1:**
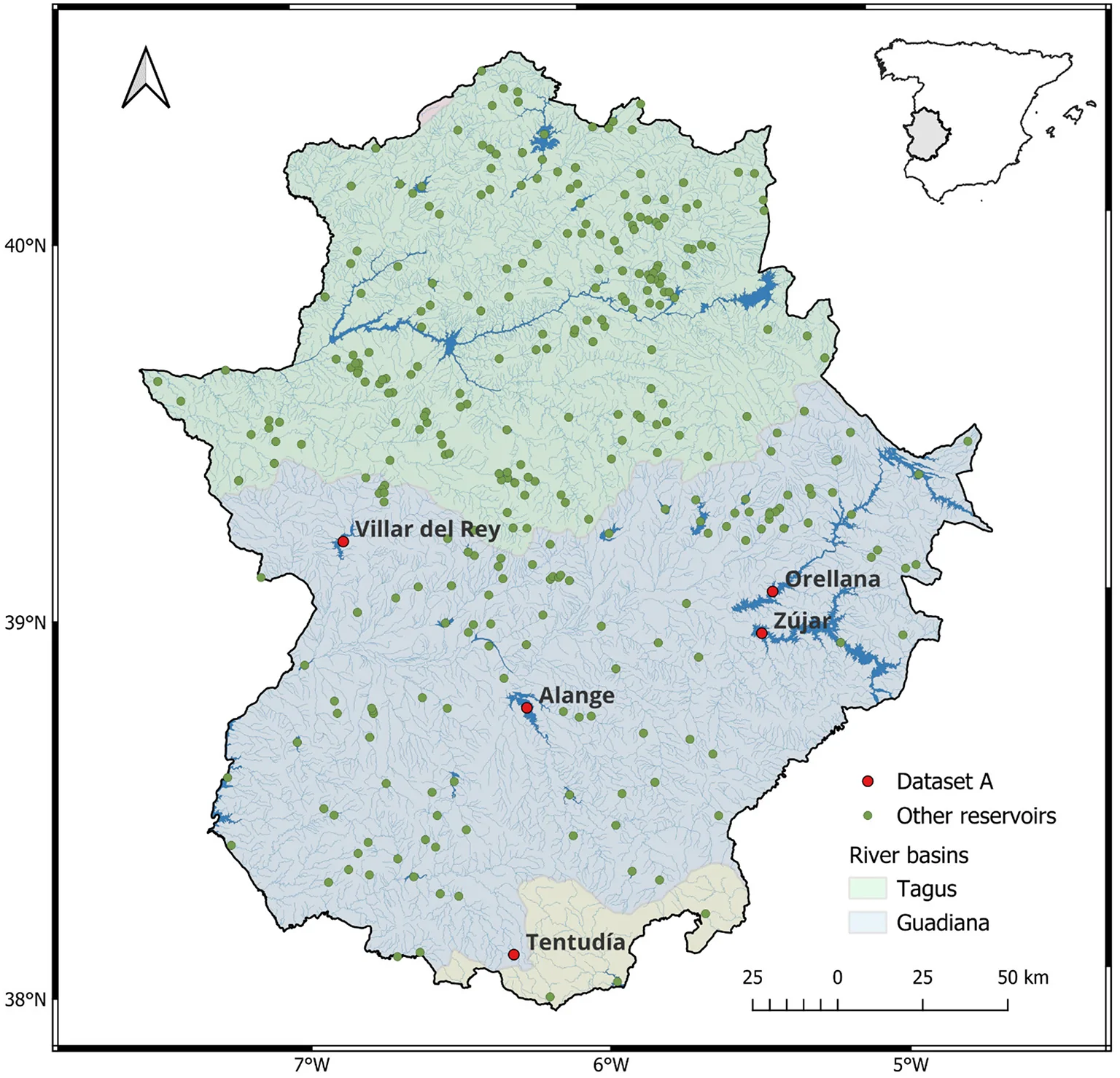
Study area. The five reservoirs of the Guadiana Basin used to calibrate and evaluate the post-correction framework.

**Table 2:** Sample size, *in-situ* Chl-a statistics, and trophic classification per reservoir.

Reservoir	*N*	Period	Min Chl-a (μg/L)	Mean Chl-a (μg/L)	Max Chl-a (μg/L)	Classification
Alange	59	2023–2025	1.69	6.89	15.92	Mesotrophic
Orellana	46	2023–2025	0.29	5.41	15.20	Mesotrophic
Tentudía	56	2023–2025	0.16	18.71	133.09	Eutrophic^†^
Villar del Rey	54	2023–2025	2.12	14.31	200.00	Eutrophic^†^
Zújar	37	2023–2025	1.27	15.49	152.77	Eutrophic^†^
**Total**	**252**					

The total matched dataset comprises 252 satellite–*in-situ* pairs. Trophic classification follows the OECD scheme (Vollenweider and Kerekes, 1982) applied to the sample-period mean Chl-a (oligotrophic ≤ 2.5, mesotrophic 2.5–8, eutrophic 8–25, hypertrophic >25 μg/L). Reservoirs marked ^†^ also exhibit episodic hypertrophic maxima exceeding 100 μg/L. The Guadiana River Basin Authority's long-term classification of all five reservoirs is mesotrophic; the sample-period means above reflect the 2023–2025 window, which includes documented bloom episodes.

### 3.2 Point-based design of the framework

A key design decision is that the framework is *point-based*: every model output is associated with a 7 × 7-pixel window (70 × 70 m on the ground) centered on the location of an *in-situ* Chl-a measurement. The correction is calibrated and evaluated only against *in-situ* samples drawn at 0.5 m depth following the Guadiana River Basin Authority's standard protocol. The framework therefore does not produce, nor is it validated to produce, spatially extrapolated Chl-a maps over the entire water body. Operationally, this is the natural unit because the only available ground truth is point-based; methodologically, it avoids spatial-autocorrelation artifacts that would arise from extrapolating across the lake surface without independent validation. Extending the correction to per-pixel inference across the full water body would require a different validation strategy and is left to future work.

*In-situ* Chl-a concentrations were obtained from the SIRA platform (https://siraguadiana.com, accessed on 24 July 2026). Sentinel-2 Level-1C images were acquired from the Copernicus Open Access Hub (https://browser.dataspace.copernicus.eu/, accessed on 24 July 2026) with cloud cover below 20%, no sun-glint contamination, and temporal proximity within ±48 h of the corresponding *in-situ* measurement. All images were resampled to 10 m by nearest-neighbor interpolation, chosen to preserve the native reflectance values rather than introduce interpolated radiances ahead of the atmospheric-correction networks, and atmospherically corrected with the three C2-Net processors in ESA SNAP at the default freshwater salinity (0.5 PSU). Because several MSI bands are natively coarser than 10 m (20 and 60 m), nearest-neighbor resampling replicates each native pixel into a block of identical 10 m pixels. Hence, the 49 pixels of a window are not all independent (a 20 m band contributes roughly a dozen distinct values, a 60 m band only one or two) and the effective sample size behind *C*_max_ is smaller than 49. The maximum is invariant to this replication, so the *C*_max_ value is unaffected, but any within-window statistic, including the 99th-percentile exclusion below, effectively draws on fewer than 49 independent values. To mitigate adjacency effects in small reservoirs, Chl-a values were extracted from the 7 × 7 pixel window using the maximum-value estimator *C*_max_ validated by Cáceres-Merino et al. (2024). This estimator is a premise of the present study rather than a parameter tuned here: Cáceres-Merino et al. (2024) established *C*_max_ as the optimal window estimator for these reservoirs, outperforming mean- and median-based alternatives, and only the resulting *C*_max_ value was retained in the matched dataset (so per-pixel statistics are not available for re-analysis here). Extreme reflectance values above the 99^*th*^ percentile were excluded. This exclusion is applied within each 7 × 7 window (*n* = 49) and *before* the maximum is taken: the single most extreme pixel is discarded and *C*_max_ is the largest of the remaining values, which guards against a lone outlier pixel driving the estimate. The resulting matched dataset comprises 252 satellite–*in-situ* pairs.

### 3.3 Symmetric 17-feature pool

The three C2-Net processor outputs differ substantially in dynamic range. C2RCC remains close to *in-situ* concentrations, whereas C2X and C2XC can overestimate by one to two orders of magnitude in optically extreme conditions. To enable a meaningful joint analysis, we rescale C2X and C2XC to the empirical moments of C2RCC:


$$\begin{array}{c} P_{r}\;\left[\mu \text{g}/\text{L}\right]=\frac{\sigma_{\text{C}2\text{RCC}}}{\sigma_{\text{P}}}\left({\text{P}}_{\text{raw}}-\mu_{\text{P}}\right)+\mu_{\text{C}2\text{RCC}} \end{array}$$ (1)

where P∈{C2X, C2XC} and the moments are computed exclusively from the training partition of each fold to avoid leakage. We note that Equation 1 is itself a modeling decision: it implicitly treats C2RCC as the scale reference. An ablation reported in Section 4.5 shows that, under Huber regression with the three *orig* features, the rescaling does not affect predictions (Huber and the upstream standardization are both linear and compose to invariance); it does, however, affect the engineered features of the symmetric pool below, whose curvature, interaction, and ratio terms are non-linear in the inputs (e.g., $P_{r}^{2}\neq P_{\text{raw}}^{2}$). We stress that Equation 1 is not intended to correct the processors' bias, which grows with concentration and is not a constant offset; it only aligns the dynamic ranges of C2X and C2XC to that of C2RCC so that the engineered features combine quantities on a common scale. The concentration-dependent bias is corrected by the downstream learner, which maps the aligned processor outputs to *in-situ* Chl-a and is free to be non-linear (MLP, Random Forest) or robust-linear (Huber). Because the deployed *orig* model is invariant to this affine step, a simple rescaling is a non-issue for that model: the correction lives entirely in the fitted regressor, not in Equation 1.

From these three base outputs we construct a symmetric pool of 17 candidate features (*FS*_17_), as determined by Equations 2–7:


$$\begin{array}{c} FS_{17}=\left\{orig,stats,inter,curv,ratio\right\} \end{array}$$ (2)

$$\begin{array}{c} orig\;\left(3\right)=\left\{\text{C}2\text{RCC},\text{C}2{\text{X}}_{r},\text{C}2\text{X}{\text{C}}_{r}\right\} \end{array}$$ (3)

$$\begin{array}{c} stats\;\left(2\right)=\left\{\text{Mea}{\text{n}}_{r},\text{Media}{\text{n}}_{r}\right\} \end{array}$$ (4)

$$\begin{array}{c} inter\;\left(3\right)=\left\{\frac{P_{i}P_{j}}{100}|i<j,P_{i},P_{j}\in orig\right\} \end{array}$$ (5)

$$\begin{array}{c} curv\;\left(6\right)=\left\{P^{2},\sqrt{\left|P\right|}|P\in orig\right\} \end{array}$$ (6)

$$\begin{array}{c} ratio\;\left(3\right)=\left\{\frac{P_{i}}{P_{j}+10^{-5}}|\left(i,j\right)\in \left\{\left(1,2\right),\left(1,3\right),\left(2,3\right)\right\}\right\} \end{array}$$ (7)

The pool is deliberately *symmetric*: nonlinear transformations and pairwise operations are applied across all three processors uniformly. This eliminates the bias that would otherwise be introduced by hand-crafting feature engineering around a favored processor or transformation.

### 3.4 Exhaustive feature search

Three regression families are used as base estimators, each representing a different inductive bias. Huber regression (Huber, 1964) is a robust linear model with a piecewise loss that transitions between squared and absolute loss at the threshold ϵ = 1.35, which makes it insensitive to the extreme outliers frequent in hypertrophic blooms. A Multi-Layer Perceptron (MLP) with two hidden layers of (50, 25) units, early stopping, and max_iter=1000 is used to capture non-linear dependencies between features. Random Forest (Breiman, 2001) with 200 trees and max_depth=15 ensembles axis-aligned splits and models regime-dependent corrections. Hyperparameter sensitivity was assessed by grid search (grids and selected values in the released code); the reported configurations lie within 1% of the optimum for Huber and MLP, and within ~6% for Random Forest, whose fully tuned optimum we examine separately in Section 4.8. The C2RCC raw output is retained as a pre-correction baseline.

The exhaustive search enumerates every non-empty subset of *FS*_17_, of which there are 2^17^−1 = 131, 071, using Huber regression with five-fold cross-validation as the screening estimator. We stress that the parsimony findings in Section 4.2 are conditional on this estimator; we comment on model-dependent caveats in Section 4.4. We recompute rescaling parameters (Equation 1) and standardization parameters inside each fold to prevent information leakage. The full enumeration is completed in under 1 h on a single CPU because each Huber fit on *n* = 252 samples takes tens of milliseconds. For comparison with conventional practice, we also evaluate three heuristic feature selectors driven by Huber regression: sequential forward selection (SFS) with neg-RMSE scoring, recursive feature elimination (RFE) on Huber coefficients, and mutual-information ranking. Each heuristic is run for a range of target subset sizes *k*∈{1, …, 12, 17}.

Reporting the single lowest-error subset from 131, 071 cross-validated candidates would be an instance of selection on the test statistic: the cross-validation error is itself a noisy random variable, and its minimum over many correlated candidates is optimistically biased, so the identity of the “winning” subset is unstable across resamples of *n* = 252. We therefore treat subset identity as a random quantity and quantify it with stability selection (Meinshausen and Bühlmann, 2010): for *B* = 500 subsamples (80% of the pairs, drawn without replacement) we re-run the selection at fixed small *k* (exhaustively at *k* = 3, and by forward sequential selection at *k* = 6; both within the plateau) with Huber regression scored by inner five-fold CV, and record the selection frequency π_*j*_ of each feature. Features selected well-above chance form the robust core; features selected near chance are read as interchangeable within the plateau rather than as signal.

### 3.5 Validation protocol

Two complementary protocols are used throughout this study. The first is a repeated five-fold cross-validation with 10 repeats, yielding 50-fold evaluations on the pooled dataset. This protocol estimates in-distribution accuracy and the variance induced by the random shuffle, and we report the mean and standard deviation across the 50 evaluations rather than a single point estimate. The second protocol is Leave-One-Reservoir-Out (LORO) validation, in which each reservoir is held out in turn while the model is trained on the remaining four. LORO enforces strict spatial separation between training and test, removes within-reservoir temporal autocorrelation, and directly simulates the operational deployment of a correction model to an unmonitored water body (Radocaj et al., 2025; Nourmohammadi et al., 2025). Bootstrap 95% confidence intervals on each held-out reservoir are obtained with 2,000 resamples.

### 3.6 Extended metric basket and its empirical validation

Most studies in this domain report some combination of RMSE, MAE, MBE, and *R*^2^. We extend this basket with three further metrics that target distinct aspects of regression behavior, then test *empirically* which of the seven carries non-redundant information on this dataset.

The seven metrics are RMSE (magnitude of error), MAE (robust magnitude), MBE (systematic bias), *R*^2^ (goodness of fit), the slope of the linear regression of predicted onto observed Chl-a (calibration drift), and the median absolute percentage error (MdAPE, with a 1 μg/L floor on the denominator to avoid divergence at very low concentrations), and the excess kurtosis of the residuals (heavy-tail diagnostic).

To test how many of the seven are needed, we evaluate 1,500 different feature subsets sampled from *FS*_17_ (stratified by subset size *k* = 1…17), compute all seven metrics on the pooled 5-fold OOF predictions, and compute the Spearman rank correlation between metrics across subsets. A caveat that should be made explicit: some correlations are partially algebraic (at the population level, $R^{2}=1-\text{RMS}{\text{E}}^{2}/\sigma_{y}^{2}$), so a ρ close to −1 between RMSE and *R*^2^ is expected and is not by itself an empirical discovery. The contribution of the analysis is to quantify residual rank disagreement between metrics whose redundancy is not algebraically forced (MAE vs. RMSE, slope vs. MBE, kurtosis vs. RMSE, and MdAPE vs. any other). All code, the matched dataset, and the trained models are available in https://github.com/vicamoresc/C2NET-PostCorrection (accessed on 24 July 2026); the repository includes a requirements.txt file specifying the Python 3.14 environment, and a fixed random seed (random_state=42) was used throughout for all stochastic components.

## 4 Results

### 4.1 Raw processor performance

We report all baselines and all machine-learning results under the same evaluation protocol: pooled RMSE on the 252 satellite–*in-situ* pairs (with repeated-CV variance bands used in Sections 4.2–4.8). Under this protocol, raw C2RCC yields an RMSE of 22.85 μg/L, C2X is substantially worse (RMSE 64.92 μg/L; mean bias error 28.3 μg/L), and C2XC is the least reliable (RMSE 172.55 μg/L; mean bias above 80 μg/L; see Figure 2). Two aggregations of the C2RCC baseline recur below and should not be conflated: the deterministic population RMSE over all 252 pairs (22.85 μg/L), and the mean of the per-fold RMSEs under repeated CV (21.24 μg/L), which is always the lower of the two because $\sqrt{\cdot }$ is concave. We compare each model against whichever baseline shares its aggregation and state the pairing at each use. These results corroborate prior findings that C2-Net processors require correction before they can support operational monitoring of small inland reservoirs (Cuartero et al., 2023; Cáceres-Merino et al., 2024; Sanchez-Perez et al., 2026; Torrecilla-Pinero et al., 2026).

**Figure 2:**
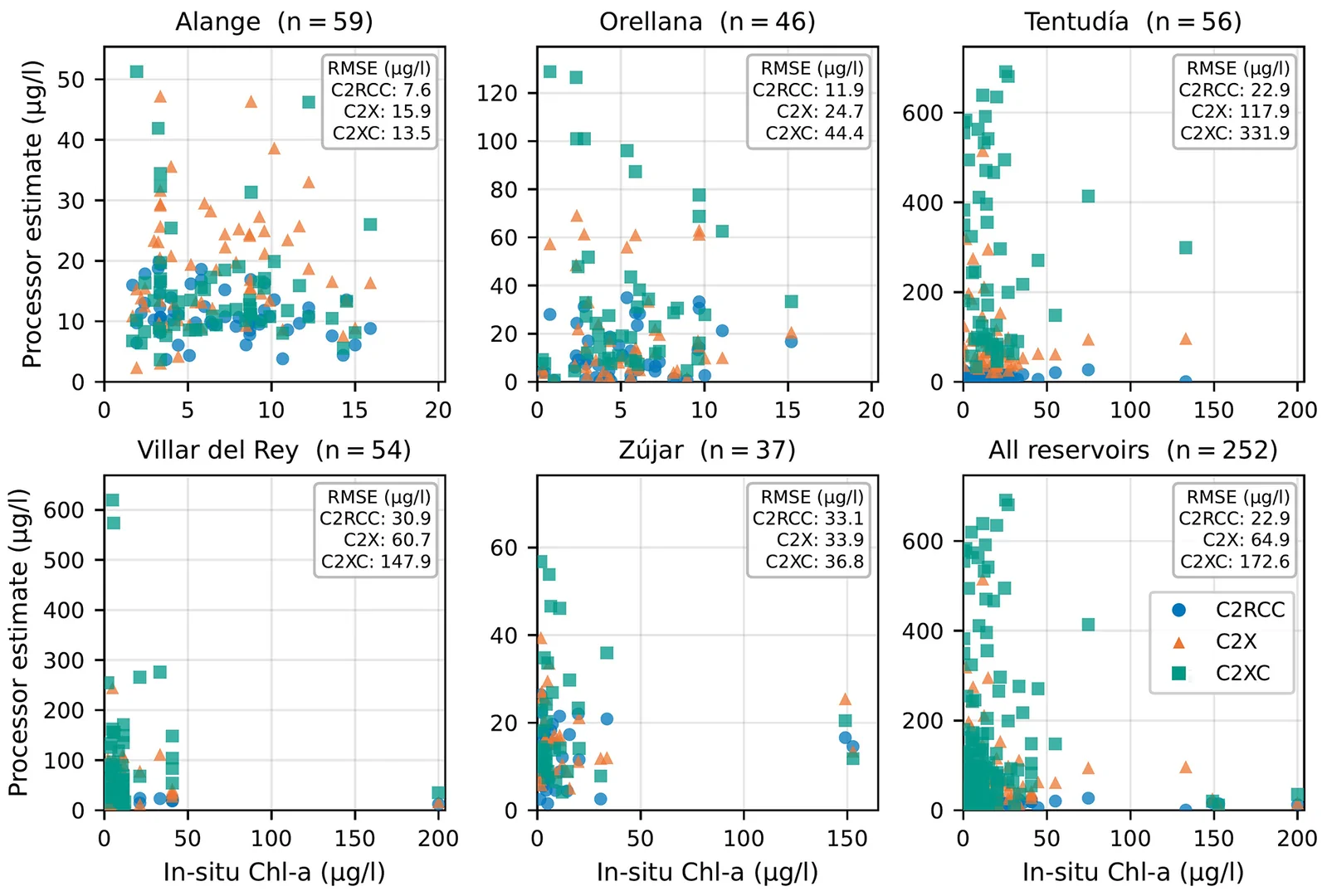
Uncorrected Chl-a estimates from C2RCC, C2X, and C2XC against *in-situ* measurements, per reservoir. Axes are scaled independently per panel (*x* to the *in-situ* range, *y* to the estimate range).

### 4.2 Exhaustive search: parsimony plateau with variance explosion at large *k*

Exhaustive enumeration of the 131,071 feature subsets shows that performance is essentially flat across a broad range of small subset sizes. Under single-seed 5-fold CV, the best one-feature model ($\sqrt{\left|\text{C}2\text{X}{\text{C}}_{r}\right|}$) achieves an RMSE of 19.00 μg/L; the best three-feature model 18.90 μg/L; the best six-feature model, $\left\{\text{C}2{\text{X}}_{r},\text{Mea}{\text{n}}_{r},\text{C}2\text{RC}{\text{C}}^{2},\text{C}2{\text{X}}_{r}^{2},\sqrt{\left|\text{C}2{\text{X}}_{r}\right|},\text{C}2{\text{X}}_{r}/\text{C}2\text{RCC}\right\}$, reaches 18.70 μg/L. This best-of-six subset happens to be composed almost entirely of C2X-derived features, but we caution against reading that composition as evidence that “the X-processor stream carries the signal”: as the stability analysis below shows, the identity of the single lowest-error subset is not stable across resamples, and this particular composition is largely a winner's-curse artifact. The question of which features robustly carry the corrective signal is answered by stability selection, not by the identity of any single optimum (Section 4.3).

To assess whether these differences are meaningful given the dataset's natural variance, we re-evaluated the best subset at each *k*∈{1, …, 17} under repeated five-fold CV (10 repeats, 50 folds in total). Figure 3 reports the mean RMSE with ±1 standard-deviation bands.

**Figure 3:**
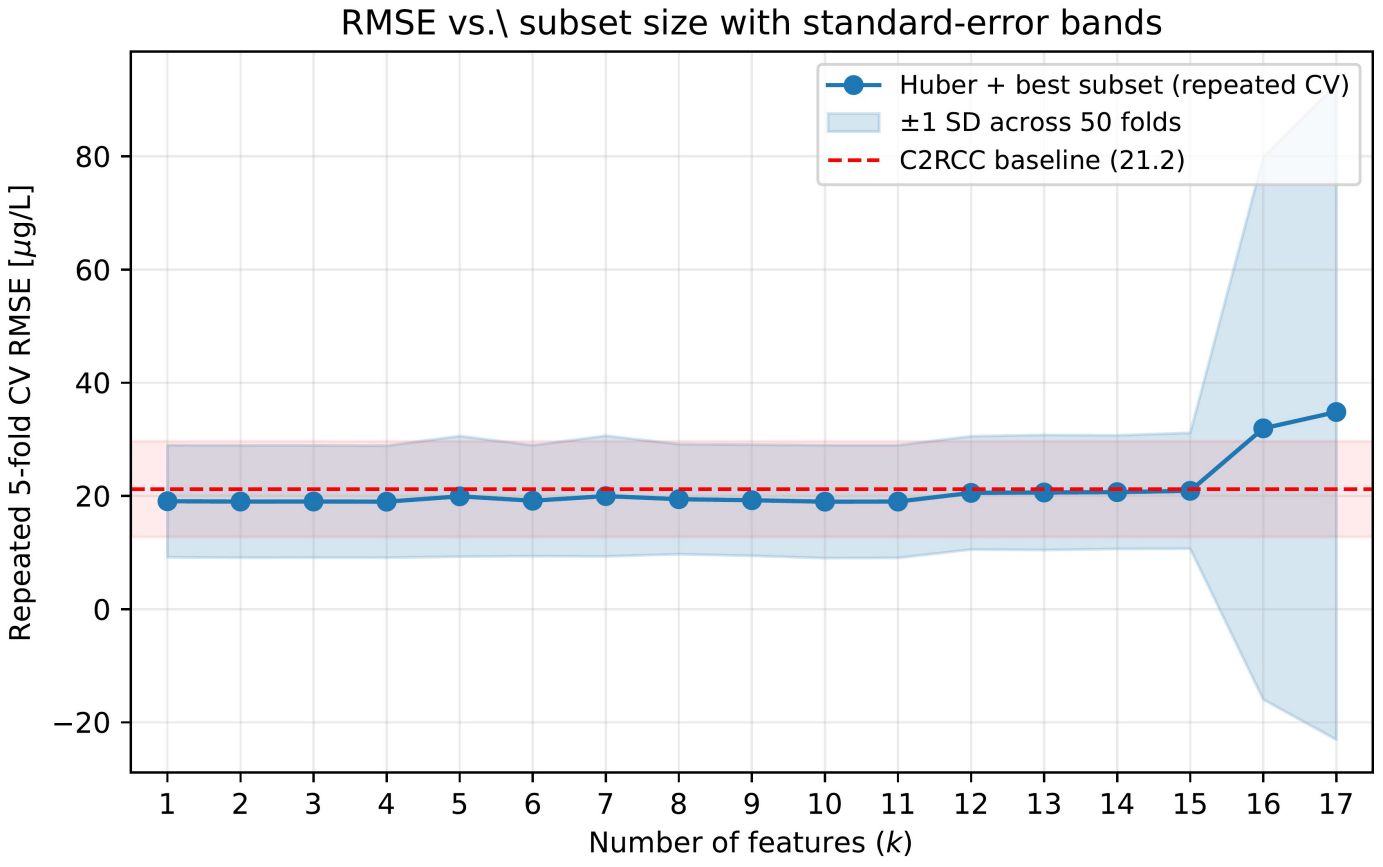
Repeated five-fold cross-validated RMSE as a function of feature subset size *k*, with ±1 standard-deviation bands across 50-fold evaluations. The red dashed line and band show the C2RCC baseline under the same protocol.

Three observations follow. First, the mean RMSE is essentially constant for *k*∈{1, …, 11} at 19.0–19.4 μg/L, with all differences an order of magnitude smaller than the across-fold standard deviation (~10 μg/L). Second, the mean RMSE rises modestly to ~20 μg/L for *k*∈{12, …, 15}. Third, for *k*∈{16, 17} the across-fold standard deviation increases sharply (47.9 and 57.9 μg/L, respectively). This is best read as a numerical-conditioning artifact rather than a finding about feature selection *per se*. At *k* ≥ 16, the design matrix contains highly correlated curvature and ratio columns, and an unregularized Huber fit occasionally becomes unstable on certain folds. This also answers why the robust Huber, rather than a penalized least-squares estimator, is the primary screening model: we ran ridge regression as the natural regularized alternative, and it does *not* damp the large-*k* variance but worsens it (full 17-feature model, repeated CV: 56.9 ± 102.3 μg/L for ridge, α = 1, against 34.8 ± 57.9 for Huber). The large-*k* blow-up is driven by the hypertrophic outliers, not by collinearity alone, so an L2 penalty on a squared loss (which remains outlier-sensitive) amplifies rather than controls it, whereas Huber's robust loss contains it. On the plateau itself, the two are equivalent (orig, ridge 19.16 vs. Huber 19.11), so regularization changes neither the plateau nor the operational message; it only fails to fix the tail. The C2RCC raw baseline lies at 21.2 μg/L under the same protocol, within ±1 SD of all model variants in the plateau. The robust takeaway is therefore not that parsimony reduces *mean* error, but that, with a non-regularized linear-robust regressor on this dataset, parsimony controls the variance of the estimator at no cost in mean accuracy. We re-emphasize that this finding is conditional on the Huber regressor. Section 4.6 examines whether the same parsimony pattern holds for non-linear estimators.

A subtlety worth flagging is that the two standard readings of feature importance disagree here, and on a plateau they need not agree: permutation importance within the *k* = 3 *orig* model ranks C2RCC first, whereas the exhaustive optima at larger *k* are dominated by C2X-derived transforms. Neither reliably indicates which features carry the signal; Section 4.3 settles that question by showing that the apparent C2X dominance is a resampling artifact.

### 4.3 Which features robustly carry the signal: stability selection

The plateau and the winner's-curse concern together imply that the identity of any single optimal subset should not be over-interpreted. To recover which features genuinely matter, we ran stability selection (Section 3.4) and recorded, across the *B* = 500 subsamples, the selection frequency π_*j*_ of each of the 17 features (Figure 4). The result is a small, sharply delineated robust core. One feature, $\sqrt{\left|\text{C}2\text{X}{\text{C}}_{r}\right|}$, dominates: it is selected in 58% of subsamples at *k* = 3 and in 98% at *k* = 6, far above any other. A second tier follows at *k* = 6: C2RCC^2^ (π = 0.72), C2XC_*r*_ (π = 0.65), and C2RCC (π = 0.62), after which frequencies fall below 0.47. Crucially, every transform of C2X on its own sits near chance (π < 0.10 at *k* = 3); C2X reaches a moderate selection frequency only in combination with another processor (the C2X_*r*_×C2RCC interaction, π = 0.30), never by itself. The C2X-dominated composition of the single best-*k* = 6 subset in Section 4.2 is therefore a winner's-curse artifact rather than evidence that the X-processor stream carries the signal on its own: the robustly-selected core draws on C2XC (through its square-root transform) and C2RCC.

**Figure 4:**
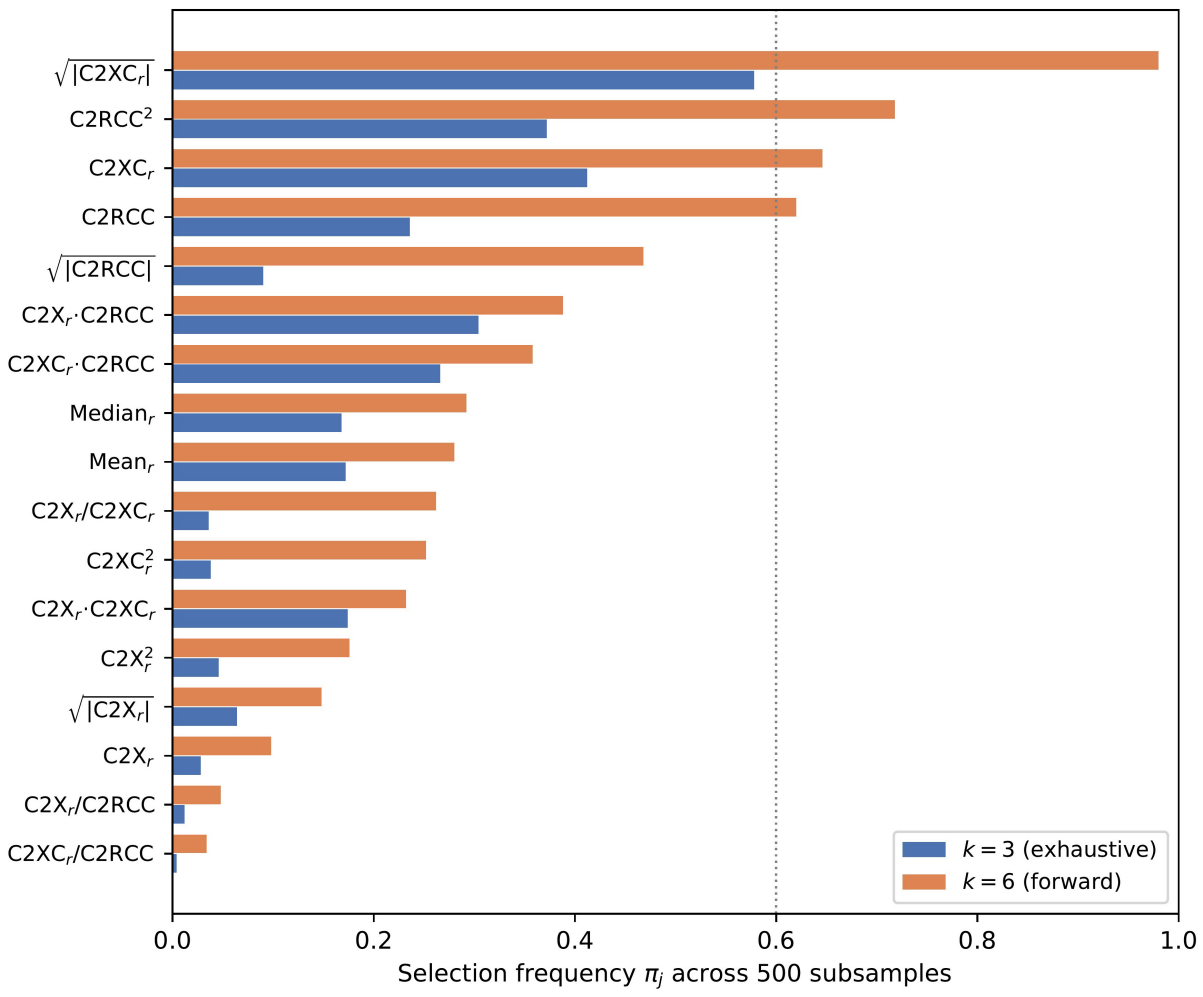
Stability selection: frequency π_*j*_ with which each of the 17 features is chosen across 500 subsamples (80% of the pairs), for the exhaustive *k* = 3 and forward *k* = 6 selectors. Only $\sqrt{\left|\text{C}2\text{X}{\text{C}}_{r}\right|}$, C2RCC^2^, C2XC_*r*_, and C2RCC are selected well-above chance; all C2X-derived features sit near the bottom.

This analysis is the rigorous form of the “which feature is best” question: it reports features by their probability of selection under resampling rather than by a single, unstable lowest-RMSE subset, and it is robust to the multiplicity of the 131, 071-candidate search in a way that reading off one optimum is not.

Because the five reservoirs are heterogeneous, unstratified 80% subsamples could in principle conflate feature stability with which reservoirs land in a given draw. Repeating the analysis with subsamples stratified by reservoir (80% drawn within each site) leaves the result essentially unchanged: the same four features form the core, with $\sqrt{\left|\text{C}2\text{X}{\text{C}}_{r}\right|}$ again selected in 98% of subsamples at *k* = 6 and every selection frequency within 0.06 of its unstratified value. The robust core is therefore a property of the features, not of the reservoir composition of the resamples.

### 4.4 Heuristic feature selection vs. exhaustive optimum

Table 3 compares the three heuristic selectors with the exhaustive optimum at the same target subset size. Three observations follow.

**Table 3:** Heuristic feature selectors vs. exhaustive optimum on *FS*_17_ with Huber regression.

*k*	Method	RMSE five-fold	RMSE LORO
1	Exhaustive	19.00	18.79
	Forward sequential	19.00	18.79
	RFE (Huber)	19.18	18.79
	Mutual information	19.18	18.79
2	Exhaustive	18.92	18.75
	Forward sequential	18.92	18.75
	RFE (Huber)	19.03	18.77
	Mutual information	19.18	18.82
3	Exhaustive	18.90	18.77
	Forward sequential	18.93	18.72
	RFE (Huber)	18.97	18.82
	Mutual information	19.02	18.77
6	Exhaustive	18.70	20.11
	Forward sequential	19.85	19.07
	RFE (Huber)	19.01	19.63
	Mutual information	18.88	18.76
10	Forward sequential	19.89	25.12
	RFE (Huber)	18.96	18.89
	Mutual information	19.59	20.73
17	Full feature set (no selection)	19.43	29.94
Reference subsets: the reported *orig* model and the stability-selected core			
3	*orig* (reported model)	19.04	18.9
4	stability-selected core	19.00	18.74

Pooled five-fold cross-validation RMSE (single seed) and Leave-One-Reservoir-Out RMSE, both in μg/L.

The exhaustive optimum is omitted at *k* = 10: at this size the plateau has already been characterized at smaller *k*, and the row is retained only to show the heuristics diverging, so the full enumeration at *k* = 10 was not computed.

First, the four methods agree at *k* = 1, 2 on the exhaustive optimum (or are within 0.1 μg/L of it). Second, at *k* = 3 all four are within 0.13 μg/L of one another but *select entirely different subsets*: the exhaustive optimum is $\left\{\text{C}2{\text{X}}_{r},\sqrt{\left|\text{C}2{\text{X}}_{r}\right|},\sqrt{\left|\text{C}2\text{X}{\text{C}}_{r}\right|}\right\}$; forward sequential picks $\left\{\text{C}2\text{X}{\text{C}}_{r}\times \text{C}2\text{RCC},\text{C}2{\text{X}}_{r}\times \text{C}2\text{X}{\text{C}}_{r},\sqrt{\left|\text{C}2\text{X}{\text{C}}_{r}\right|}\right\}$. Both lie on the same performance plateau. At *k* = 6 this over-fitting becomes explicit: the exhaustive optimum attains the lowest cross-validated RMSE (18.70 μg/L) yet the highest LORO RMSE (20.11) of the four methods, worse than every heuristic at that size, a direct instance of the selection-on-the-statistic effect that motivates the stability-selection analysis of Section 4.3. Third, as *k* grows the heuristics diverge sharply: forward sequential at *k* = 10 has LORO RMSE = 25.1 μg/L, and the full 17-feature model has 29.9 μg/L, the worst of all configurations tested. The exhaustive search makes both the plateau and its breakdown visible; heuristic selection alone cannot distinguish between the two.

From Section 4.8 onward we report the deployed model on the *orig* feature set (the three raw processor outputs, without engineered transforms), and the reference rows of Table 3 justify that choice. Under the same protocol, *orig* (five-fold 19.04, LORO 18.99 μg/L) is statistically indistinguishable from the stability-selected core $\left\{\text{C}2\text{RCC},\text{C}2\text{X}{\text{C}}_{r},\text{C}2\text{RC}{\text{C}}^{2},\sqrt{\left|\text{C}2\text{X}{\text{C}}_{r}\right|}\right\}$ (19.00, 18.74) and from every small-*k* selector: all sit on the same plateau, with differences an order of magnitude below the across-fold standard deviation. The stability analysis is therefore a *diagnostic* that identifies which features robustly carry the corrective signal, not a prescription for a different deployed model; we report *orig* because it is the most parsimonious and interpretable member of that plateau (three physical outputs, no engineered features, and, for the linear model, exact invariance to the rescaling of Equation 1), whereas the core confirms that the engineered transforms add nothing deployable beyond it.

### 4.5 Ablation of the rescaling step

The fold-wise rescaling of Equation 1 is irrelevant to the linear *orig*-only model (Huber with a learned intercept is invariant to linear input transforms) but not to the engineered pool *FS*_17_, whose curvature, interaction, and ratio features are non-linear in the inputs. Removing it inflates the full-pool RMSE from 35 to 46 μg/L and the best-*k* = 6 RMSE from 19 to 39, with much wider fold variance, confirming that the rescaling carries real information once engineered features are admitted; computing it fold-wise rather than on the full dataset costs almost nothing (34.8 vs. 37.4 μg/L), so we retain the fold-wise version for protocol cleanliness.

### 4.6 Model-dependence of the parsimony pattern

The parsimony plateau is specific to Huber's linear-robust loss. Repeating the RMSE-vs.-*k* analysis with Random Forest (exhaustive for *k* ≤ 3, 200 sampled subsets per *k* above) yields an essentially flat curve at ~21 μg/L across all *k*, with no plateau and no variance blow-up at large *k*: the tree ensemble absorbs the correlated curvature and ratio columns without the numerical conditioning that destabilizes the unregularized linear fit at *k* ≥ 16. Subset size is therefore not a useful design choice under Random Forest, and the parsimony finding does not generalize to estimators that control variance intrinsically. In the plateau region, the two are close (~19 vs. ~21 μg/L), consistent with the estimator-equivalence reported in Section 4.8.

Figure 5 overlays the two curves and makes the contrast direct. Two features distinguish them. First, the Huber curve rises, and its variance band widens sharply for *k* ≥ 16, whereas the Random Forest band stays near ±8 μg/L at every *k*: the ensemble's intrinsic variance control removes both the incentive for parsimony and the large-*k* instability. Second, the curves cross near *k* = 15, with Huber holding a small mean advantage inside the plateau and losing it once the correlated columns dominate. This is the sense in which the two estimators tell different stories about feature-set size, and it is why we treat the parsimony plateau as conditional on a linear-robust loss rather than as a property of the data alone. Regularization alone does not account for the difference: as Section 4.2 shows, ridge regression does not damp the large-*k* variance (it worsens it, the blow-up being outlier- rather than collinearity-driven), so the operative distinction is between robust and non-robust losses, and Random Forest controls the variance through its ensembling rather than through any penalty.

**Figure 5:**
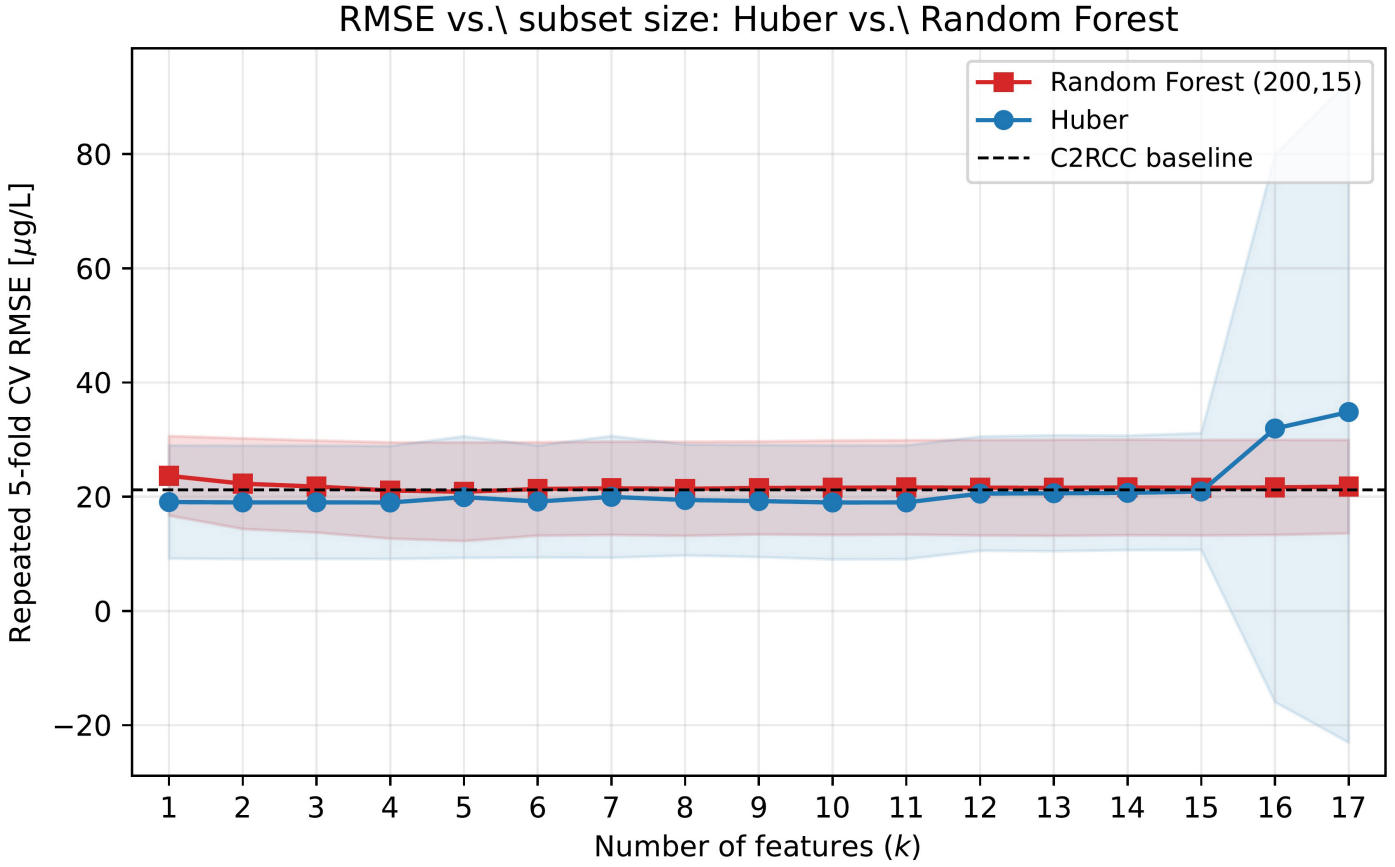
RMSE-vs.-*k* curves for Huber regression and Random Forest under repeated five-fold CV. Shaded bands are ±1 standard deviation across 50-fold evaluations; the horizontal line is the raw C2RCC baseline. Huber shows the parsimony plateau and a sharp variance increase at *k* ≥ 16; Random Forest is flat throughout.

### 4.7 Empirical validation of the extended metric basket

Across the 1,500 evaluated subsets, the seven metrics are largely redundant. MAE tracks RMSE (ρ = 0.95), residual kurtosis tracks it inversely (ρ = −0.97), MBE overlaps with the calibration slope (ρ = 0.81), and the ρ(RMSE, R^2^) = −1.00 tie is algebraic rather than empirical. MdAPE is the sole exception, correlating with each of the other six at |ρ| ≤ 0.30. It is the only genuinely orthogonal axis, contributing a relative-error signal that matters when targets span orders of magnitude, as inland-water Chl-a does. Its 1 μg/L floor affects only 3.2% of the samples (8 of 252), and because MdAPE is a median, these tail samples do not move it: the Huber MdAPE is 54.2% with or without the floor. Based on this single dataset, we therefore suggest reporting MdAPE alongside RMSE, *R*^2^, and MBE, while noting that the generality of this recommendation beyond the present case is a motivated hypothesis rather than an established result.

### 4.8 Per-reservoir correction performance

Using the parsimonious *orig* feature set, Table 4 reports the per-reservoir performance of Huber, MLP, and RF against the uncorrected C2-Net baselines, under repeated five-fold CV with 10 repeats. Comparing these means with the previously published single-seed numbers reveals just how unstable point estimates can be on small per-reservoir samples: for example, for Villar del Rey, Huber's single-seed 70/30 RMSE was 8.26 μg/L while the repeated-CV mean is 19.6 ± 19.8 μg/L. We recommend the repeated-CV numbers as the more honest point of reference.

**Table 4:** Per-reservoir correction performance using *orig* features under repeated five-fold CV (10 repeats × 5 folds = 50 fold evaluations).

Reservoir	*N*	Huber RMSE	MLP RMSE	RF RMSE	C2RCC raw RMSE
Alange	59	4.02 ± 0.90	4.22 ± 1.02	4.45 ± 1.05	5.86
Orellana	46	3.18 ± 0.95	3.39 ± 1.04	3.71 ± 1.18	7.60
Tentudía	56	18.52 ± 10.46	19.71 ± 11.12	17.94 ± 9.85	34.09
Villar del Rey	54	19.59 ± 19.76	21.34 ± 19.95	16.85 ± 12.14	17.41
Zújar	37	25.31 ± 23.96	25.92 ± 23.61	23.57 ± 21.40	40.22
All (pooled)	252	19.11 ± 9.88	19.77 ± 9.73	21.08 ± 8.42	21.24

RMSEs in μg/L. Model columns are mean ± standard deviation across the 50 folds; the C2RCC column reports the per-fold mean (21.24 μg/L pooled), the like-for-like baseline for these columns (see Section 4.1).

Across the pooled dataset, Huber regression has the lowest per-fold mean RMSE (19.11 μg/L): a ~16% reduction against the population baseline (22.85) and a ~10% reduction against the like-for-like per-fold-mean baseline (21.24), the model gain being in either case well within the variability of any individual fold. The per-reservoir picture varies by site: at Orellana the correction approximately halves the RMSE (7.60 to 3.18 μg/L, a 58% reduction) and at Alange it reduces RMSE by about 31% (5.86 to 4.02); at Tentudía the reduction is ~45%, at Villar del Rey roughly a tie with C2RCC, and at Zújar Huber's per-fold-mean RMSE (25.31 μg/L) is lower than raw C2RCC (40.22) but with a standard deviation across folds (±23.96) almost as large as the mean itself, driven by a small number of extreme bloom samples discussed in Section 5.2.

Random Forest needs a separate comment. Under per-fold-mean aggregation, it (21.08 μg/L) is essentially indistinguishable from raw C2RCC (21.24 μg/L); it improves over C2RCC at each reservoir, but the gains shrink to within the margin once aggregated because RF's predictions on rare hypertrophic samples inflate the pooled metric. To rule out under-tuning, we re-tuned RF with the same effort as Huber and MLP, using a grid over the number of trees, maximum depth, and the minimum samples per split and per leaf. Its optimum (500 trees, max_depth=5) reaches 20.11 μg/L under single-seed five-fold CV and 19.81 under repeated CV, an improvement over the same-protocol configuration but still not below Huber (19.11). A fully-tuned tree ensemble, therefore, does not overtake the robust linear model on this dataset.

Per-sample squared errors pooled across the 50 repeated-CV folds are not independent: each sample serves as a test point in roughly 10 folds, and the training partitions overlap heavily, so a naive Wilcoxon test on these errors would inflate the effective sample size and underestimate the *p*-values. We therefore assess significance with the Nadeau–Bengio corrected paired *t*-test on the 50 per-fold RMSEs (Nadeau and Bengio, 2003), which adjusts the variance for this train/test overlap. We apply it in two ways. First, against the pre-correction baseline: the Huber correction improves on raw C2RCC by a statistically significant margin (*p* = 0.047; per-fold mean 19.11 vs. 21.24 μg/L), whereas the same test does not reach significance for MLP (*p* = 0.16) or the re-tuned Random Forest (*p* = 0.09) against C2RCC. This is the comparison that underwrites the study's “modest but real” framing, and it is the strongest model, Huber, that clears the bar. Second, among the estimators themselves, none of the three pairwise differences is significant: *p* = 0.18 for Huber vs. MLP, *p* = 0.21 for Huber vs. the re-tuned Random Forest, and *p* = 0.94 for Random Forest vs. MLP. On this dataset, therefore, no estimator holds a statistically significant accuracy advantage over the others, and the practical preference for Huber rests on its interpretability and lower computational footprint rather than on a measurable accuracy gap.

Following the reporting template in Section 4.7, Table 5 presents the full metric basket for the pooled model under the same per-fold-mean aggregation. Beyond RMSE, the correction cuts MAE from 12.1 to 8.2 μg/L and MdAPE from 90% to 54%, and lifts *R*^2^ from −0.62 to −0.03 (still marginally negative because a few hypertrophic folds dominate the pooled variance, so no model beats the mean predictor in *R*^2^ terms on the pooled set). The bias signal is the most informative addition for a post-correction study: Huber slightly under-predicts (MBE −4.1 μg/L) whereas the tree ensemble is almost unbiased (MBE +0.5) at the cost of a higher RMSE, a trade-off that reporting RMSE alone would hide.

**Table 5:** Pooled metric basket for the correction models and the raw C2RCC baseline, *orig* features, under repeated five-fold CV (means across the 50 folds; same aggregation as Table 4).

Model	RMSE	MAE	*R* ^2^	*MBE*	*MdAPE (%)*
Raw C2RCC	21.24	12.05	−0.62	+2.03	89.5
Huber	**19.11**	**8.22**	**−0.03**	−4.09	**53.9**
MLP	19.77	8.90	−0.16	−3.62	61.2
RF	21.08	10.27	−0.58	**+0.45**	62.4

MBE and MdAPE are the relative-error and bias axes that Section 4.7 identifies as non-redundant.

RMSE, MAE, and MBE in μg/L; MdAPE in %. Bold marks the best value per column.

Figure 6 makes the correction visible directly, contrasting raw C2RCC with the Huber-corrected estimate on a single representative out-of-fold split. It removes C2RCC's scatter at low concentrations but also compresses predictions into a narrow 5–18 μg/L band that visibly under-predicts the hypertrophic events; its single-split pooled RMSE (21.5 μg/L) sits above the repeated-CV mean of 19.11, consistent with the single-seed instability noted above.

**Figure 6:**
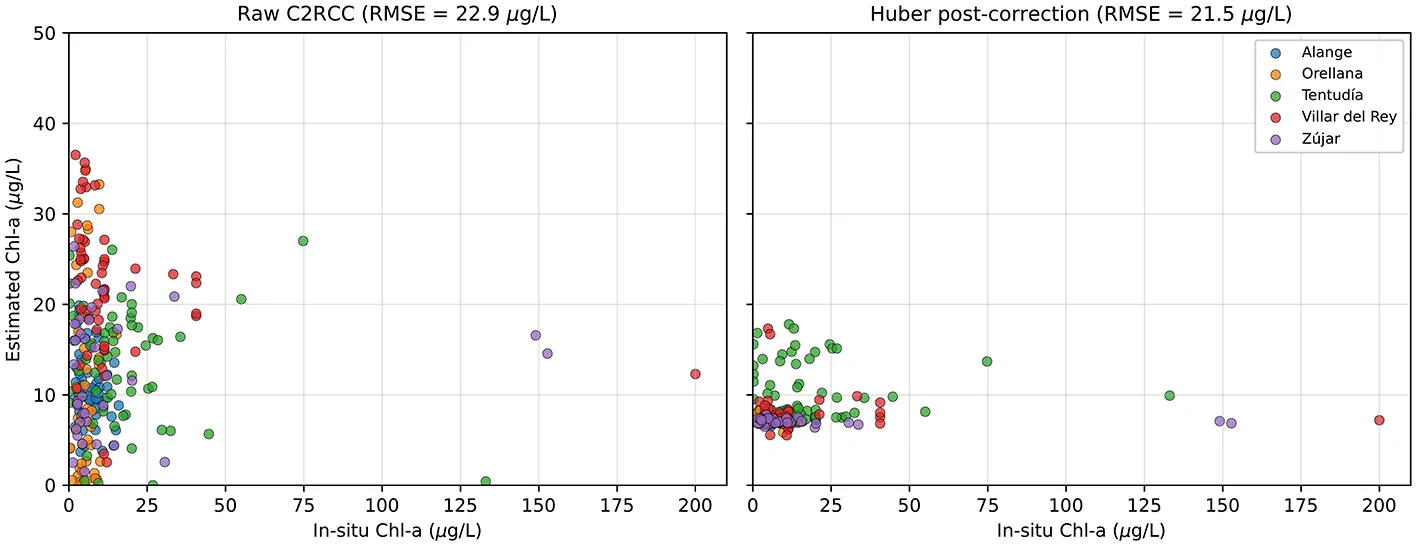
In-distribution predicted-vs.-observed Chl-a on a single five-fold out-of-fold split (seed 42): raw C2RCC **(left)** vs. Huber post-correction **(right)**, color-coded by reservoir. Axes are scaled independently (*x* to the *in-situ* range, *y* to 0–50 μg/L).

### 4.9 Spatial transferability: LORO

Table 6 reports two aggregations that are commonly conflated in this literature: the unweighted mean of per-reservoir RMSEs (18.99 μg/L) and the pooled RMSE over all 252 LORO out-of-fold predictions (21.92 μg/L). They differ by 16% because the large, low-error reservoirs (Alange, Orellana) pull the unweighted mean down, whereas the pooled metric weights every sample equally and is dominated by the hypertrophic events at Tentudía, Villar del Rey, and Zújar. The like-with-like comparison is pooled against pooled. Huber's LORO RMSE (21.92) vs. its repeated-CV RMSE (19.11, Table 4) shows a ~15% degradation under spatial separation, and the per-reservoir-mean comparison (18.99 vs. 14.12) strengthens the same conclusion to 34%. The correction therefore survives spatial separation (its LORO RMSE stays below raw C2RCC at 22.85) but generalizes meaningfully worse than within-reservoir random splits. We accordingly do not read the near-equality of unweighted-mean LORO and pooled CV as evidence of strong transferability, since that equality was an aggregation artifact. Both degradation figures should nonetheless be read as approximate. With only five held-out reservoirs, and given the wide per-reservoir bootstrap intervals of Table 6 (for example Zújar [6.03, 53.35] μg/L), the ~15% and ~34% values sit well within the sampling noise, so we report both but treat them as order-of-magnitude rather than precise transferability estimates. The Nadeau–Bengio correction we apply to the estimator comparison (Section 4.8) is not the appropriate tool for the LORO–CV gap, because it corrects the variance of *repeated* random resampling, whereas LORO is a single deterministic partition into five spatial folds with no repeats, leaving no resampling variance to correct and only five paired points. The bootstrap confidence intervals of Table 6 are the honest expression of that uncertainty, and they already show that the transferability figure is imprecise.

**Table 6:** Leave-One-Reservoir-Out validation for Huber regression with *orig* features.

Held-out reservoir	*N*_test_	RMSE (μg/L)	95% CI
Alange	59	3.75	[3.24, 4.26]
Orellana	46	4.50	[3.84, 5.11]
Tentudía	56	24.13	[12.92, 35.02]
Villar del Rey	54	28.28	[7.71, 47.11]
Zújar	37	34.30	[6.03, 53.35]
Mean of per-reservoir RMSEs (all 5)	—	18.99	
Pooled RMSE over all 252 LORO predictions	252	21.92	

RMSE in μg/L. Confidence intervals from percentile bootstrap (2,000 resamples). Both per-fold mean and pooled OOF RMSE are reported for the aggregate row.

The bootstrap 95% CIs are wide for the three reservoirs with hypertrophic events (Tentudía, Villar del Rey, and Zújar), reflecting the fact that the LORO test partition for each contains 37–56 samples and a handful of extreme values drive the metric. CIs at Alange and Orellana are tight (±0.5–0.6 μg/L); we do not, however, claim this matches laboratory analytical uncertainty, only that the model is stable within those two specific reservoirs.

Figure 7 shows the LORO predicted-vs.-observed scatter, color-coded by held-out reservoir.

**Figure 7:**
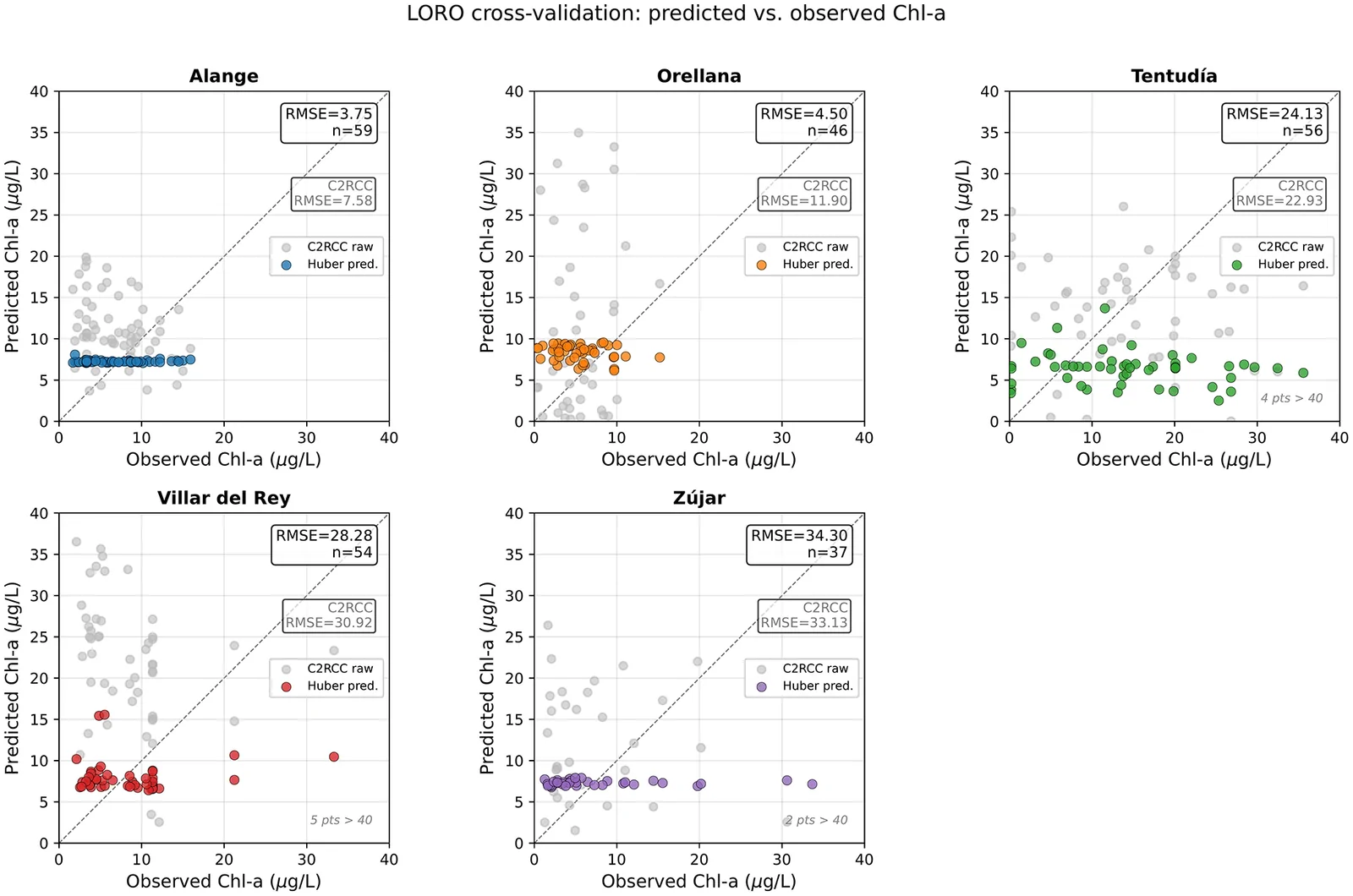
LORO predicted vs. observed Chl-a. Points are color-coded by held-out reservoir. Dashed line is the 1:1 relationship.

## 5 Discussion

### 5.1 What the exhaustive search adds

The exhaustive enumeration is a *diagnostic* for the heuristic selectors routinely deployed in this area, not a proposed algorithm, and three things it reveals would be invisible under heuristic search alone. First, the RMSE landscape is flat for *k*∈{1, …, 11} once across-fold variance is accounted for Figure 3, so heuristics reach different subsets at near-identical error, and any single “optimum” is arbitrary on this plateau; reports of a unique optimum in similar studies should be treated with caution unless the across-fold variance is given. Second, the variance explodes for *k* ≥ 16 (SD 57.9 μg/L, over five times the mean RMSE), a pattern a greedy heuristic cannot see. Third, the symmetric construction of *FS*_17_ is the prerequisite for the stability result: an asymmetric pool engineered around C2RCC could never have surfaced $\sqrt{\left|\text{C}2\text{X}{\text{C}}_{r}\right|}$, the most stably-selected feature, decoupling the conclusion from the engineer's prior about which processor matters.

Two questions follow about that feature. The first concerns why this transform set (squares, square roots, and pairwise products and ratios) rather than, say, log-ratios. It is a deliberately minimal, symmetric non-linear basis applied identically to all three processors (squares for curvature, roots to compress heavy tails, and products and ratios for interactions); it is not exhaustive, and other bases could be substituted. The point of the symmetric construction is that no processor or transform is privileged a priori, so that stability selection, not the analyst, decides what survives. The second question is whether $\sqrt{\left|\text{C}2\text{X}{\text{C}}_{r}\right|}$ carries real signal or is an artifact of that basis. Its selection has a direct statistical interpretation. C2XC is by far the noisiest processor (raw RMSE 172 μg/L, overestimating by one to two orders of magnitude in optically extreme water; Table 1, Figure 2), and the square root is the classical variance-stabilizing transform for such heavy-tailed quantities, compressing C2XC's inflated tail back toward the concentration scale so that its residual information becomes usable. The surviving transform is exactly the one that stabilizes the variance of the noisiest processor, not an arbitrary artifact. This is also the concrete insight the stable core adds beyond the modest accuracy gain: it localizes the corrective signal to C2RCC's level and C2XC's variance-stabilized tail, and shows that the C2X stream, despite dominating the single lowest-error subset, carries no robust information, a point a headline RMSE alone would not reveal.

Two caveats apply: exhaustive enumeration is feasible only because *p* = 17 and *n* = 252 and does not scale. Explaining *why* the manifold of equivalent corrections takes its shape is beyond our scope (its model-dependence is shown with Random Forest in Section 4.6). The metric-correlation analysis serves the same diagnostic intent: since the *R*^2^/RMSE tie is algebraic, the empirical content is that MAE tracks RMSE and MBE overlaps with slope, leaving MdAPE as the only genuinely orthogonal axis, to be reported alongside RMSE rather than instead of it.

### 5.2 The Zújar anomaly: two sample-level out-of-distribution events

At Zújar, the correction improves over raw C2RCC in aggregate (repeated-CV RMSE 25.31 vs. 40.22; LORO 34.30 vs. 40.22), but the gain is low-confidence: the LORO bootstrap CI is [6.03, 53.35] μg/L and the across-fold SD (±23.96) nearly equals the mean, both driven by a few extreme samples. Two *in-situ* events exceeding 149 μg/L in January 2024 (coincident with a 200 μg/L reading at Villar del Rey) are the cause: for both dates, all three processors agree on Chl-a below 25 μg/L (inter-processor CV 8%–17%), so the satellite simply does not see what the sensor measured.

We read this as sample-level, not reservoir-level: the bulk of Zújar behaves like the rest of the dataset, and excluding only these two events drops its LORO RMSE from 34.30 to 7.79 μg/L (a diagnostic of how sensitive aggregate metrics are to extremes, not a headline). The direction of the disagreement is itself informative. Because *C*_max_ selects the brightest pixel in the window, a patchy surface bloom would tend to inflate at least one processor's estimate; instead, all three agree on low values with a tight inter-processor spread, which argues against surface patchiness and for a signal the passive sensor cannot see. A candidate mechanism is a sub-surface chlorophyll maximum invisible to passive optical sensing (Cullen, 1982), plausible under winter stratification, but sampling or laboratory error and bloom advection are equally consistent; discriminating them needs depth-resolved CTD/fluorometry, duplicate sampling, or Sentinel-3 OLCI cross-checks we do not have. Operationally, this implies that an unsupervised outlier-detection layer should flag samples where the three processors are jointly inconsistent with the *in-situ* record.

### 5.3 Comparison with existing approaches

Direct numerical comparison with published inland-water Chl-a studies is of limited value, since they span very different geographies, concentration ranges, and dataset sizes; the validation protocol matters more than the reported RMSE. For context, our repeated-CV RMSE (19.1 μg/L) lies in the 15–20 μg/L range of multi-lake Random Forest and XGBoost retrievals (Saberioon et al., 2020; Wang et al., 2022; Santos et al., 2024), and our spatial-CV RMSE (18.99 as a per-reservoir mean, 21.92 pooled) is comparable despite enforcing the stricter out-of-distribution protocol those studies do not; single-site CNNs report much lower errors (King et al., 2025) only because they train and test on the same water body. The most directly comparable study is our group's breadth-first companion (Torrecilla-Pinero et al., 2026) (32 reservoirs, 94 scenes, −35% vs. the best baseline under spatial CV); its percentage gains are not directly comparable to ours owing to a different baseline-protocol pairing, but together the two show the post-correction paradigm working both across many sites and, here, under deeper temporal sampling and the LORO stress test, albeit with modest pooled gains.

The post-correction angle differentiates our approach from most cited works. Rather than discarding atmospheric correction and learning from radiances, we keep the physical information already extracted by C2-Net and correct its systematic biases. The framework can therefore be re-applied at marginal cost whenever the underlying processors are updated, and the simplicity of the model (Huber regression with three inputs) makes it auditable in operational pipelines, an increasingly valued property in regulatory monitoring (Ansari et al., 2025; Mohan et al., 2025).

### 5.4 Limitations

Several limitations bound the conclusions of this work. The first concerns dataset size: the 252 matched pairs are sufficient for the modest-capacity models employed here, but they limit the representation of extreme trophic conditions and explain part of the LORO variance at Tentudía and Villar del Rey. With 37–59 samples per reservoir, per-reservoir point estimates have wide bootstrap confidence intervals (Table 6) and should not be over-interpreted; the 32-reservoir, 94-scene dataset analyzed in our group's parallel work (Torrecilla-Pinero et al., 2026) provides the natural complementary axis (more sites, fewer matches per site), and merging both datasets into a unified calibration corpus is the obvious next step. A second limitation is the cost of the exhaustive sweep: full enumeration of *FS*_17_ is cheap with Huber regression (~1 h on a single CPU) but expensive with Random Forest (extrapolating from our per-fit timings, ~18 h for the full enumeration). We therefore evaluated Random Forest in Section 4.6 using a hybrid scheme (full enumeration for *k* ≤ 3 and 200 random subsets per *k* for *k* ≥ 4) rather than a true exhaustive sweep. The qualitative finding that Random Forest yields a flat RMSE-vs.-*k* curve without a parsimony plateau is robust to the sampling choice, but a quantitative comparison of the absolute best subset between Huber and Random Forest would require the full ~18 h sweep we did not run.

The temporal matching window also deserves mention: the ±48-h interval between satellite overpass and *in-situ* sampling can mask rapid bloom dynamics (Cáceres-Merino et al., 2024), and tighter matching would require denser field campaigns that contradict the operational motivation of satellite monitoring. Related to this, Sentinel-2 MSI is multispectral with native resolutions of 10–60 m per band; the 7 × 7 extraction window reduces the effective spatial resolution at the calibration unit to 70 m, and the framework is, as stated in Section 3.2, point-based by design: it does not produce or validate spatially extrapolated Chl-a maps. Extending the correction to per-pixel inference across the full water body is a structurally different problem requiring additional ground truth or a calibrated assumption of within-lake homogeneity at intermediate scales. Finally, the framework was calibrated and validated entirely within the Guadiana Basin, so cross-basin transfer experiments are required to assess generalization across broader biogeographic gradients (Ratte-Fortin et al., 2026; Xu et al., 2026). It is also worth distinguishing what this study demonstrates from what remains plausible. Within the Guadiana Basin, the correction improves modestly but significantly on raw C2RCC (Section 4.8), the corrective signal localizes to a small stability-selected feature core (Section 4.3), and performance survives spatial separation to unseen reservoirs of the basin under LORO, albeit with a measurable and imprecisely estimated degradation (Section 4.9). What we cannot yet claim, and leave as an open operational question, is transfer to reservoirs in other basins whose optical water types, atmospheric regimes, and C2-Net error structures differ. The methodological products are nonetheless basin-independent by construction, since the exhaustive-vs.-heuristic audit, stability selection in place of a single “best” subset, the cross-validation-corrected significance test, and LORO as a minimum transferability check are procedures rather than parameters, and apply unchanged wherever a post-correction layer is fitted.

### 5.5 Future directions

Three directions extend the present work naturally. The first is integrating auxiliary variables (water temperature, wind speed, and Secchi depth) that could constrain predictions during extreme events; the open question is whether these gains survive under LORO. The second is per-sample prediction uncertainty: we currently report bootstrap confidence intervals on aggregate RMSE, which describe sampling variability in the test-set metric but not per-sample prediction intervals. Conformal prediction (Angelopoulos and Bates, 2023) provides distribution-free per-sample prediction intervals with calibrated coverage and is the natural complement to the present pipeline, since bootstrap CIs and conformal intervals solve different problems (aggregate sampling variability vs. per-sample uncertainty at deployment time). The third is a trophic-regime-aware cascade model that would specialize the correction to oligotrophic, mesotrophic, and hypertrophic regimes, with the C2-Net outputs themselves driving the regime classification. Extending MLP and gradient-boosting estimators following the protocol introduced in Section 4.6 would also close out the model-dependence question.

## 6 Conclusions

This case study evaluated a data-driven post-correction framework for the C2-Net atmospheric correction processors applied to Sentinel-2 imagery. The empirical bottom line is that the gain over raw C2RCC under same-protocol comparison is small (~10%–16% pooled RMSE reduction depending on aggregation), though statistically significant for the Huber model under a cross-validation-corrected test (*p* = 0.047); among the three estimators themselves, no significant accuracy advantage exists. The framework's contribution is therefore methodological rather than a headline accuracy improvement.

Several conclusions follow. First, exhaustive enumeration of the symmetric 17-feature pool reveals a broad plateau of equivalent solutions across small subset sizes (Huber): heuristic selectors disagree on which specific subset to pick within this plateau, the variance increase at *k* ≥ 16 is best read as a numerical-conditioning artifact, and the parsimony pattern does not survive substitution of Huber by Random Forest (Section 4.6). Because the single lowest-error subset is a winner's-curse artifact on this plateau, we identify the features that robustly carry the signal by stability selection instead: a small core led by $\sqrt{\left|\text{C}2\text{X}{\text{C}}_{r}\right|}$ (selected in 98% of subsamples at *k* = 6), with the C2X-derived transforms that dominate the single best subset selected only near chance. Second, the metric-correlation study shows that *R*^2^ and RMSE are algebraically tied, MAE and residual kurtosis add little diagnostic information beyond RMSE on this dataset, and MdAPE provides the only genuinely orthogonal axis (relative error). Third, under like-with-like aggregation, the LORO RMSE (21.92 μg/L pooled across the five held-out reservoirs) is approximately 15% higher than the in-distribution repeated-CV RMSE (19.11): the correction does survive spatial separation, but the deterioration under LORO is meaningful, and the earlier “near-equality” reading of the unweighted-mean LORO statistic was an aggregation artifact. Finally, two extreme bloom samples at Zújar dominate that reservoir's residual variance; the satellite-radiometer hypothesis (sub-surface chlorophyll maximum invisible to passive optical sensing) is consistent with the data but not proved by them, and reframes the Zújar finding as a sample-level out-of-distribution case rather than a reservoir-level failure of the method.

The combined methodological suggestion, abstracted from this case, is a small reporting template: an RMSE-vs.-*k* curve with across-fold variance bands rather than a single optimum subset; stability selection rather than the identity of a single lowest-error subset when reporting which features matter; at least one relative-error metric alongside the absolute-error triple; like-with-like aggregation when comparing CV and spatial-CV protocols; and LORO as a minimum check on any transferability claim in geospatial machine learning. We frame this as a hypothesis motivated by one dataset rather than an established prescription; its value across other basins and processors remains to be tested. These elements are, in any case, inexpensive to add to existing pipelines.

## Data Availability

The datasets presented in this study can be found in online repositories. The names of the repository/repositories and accession number(s) can be found in the article/supplementary material.
